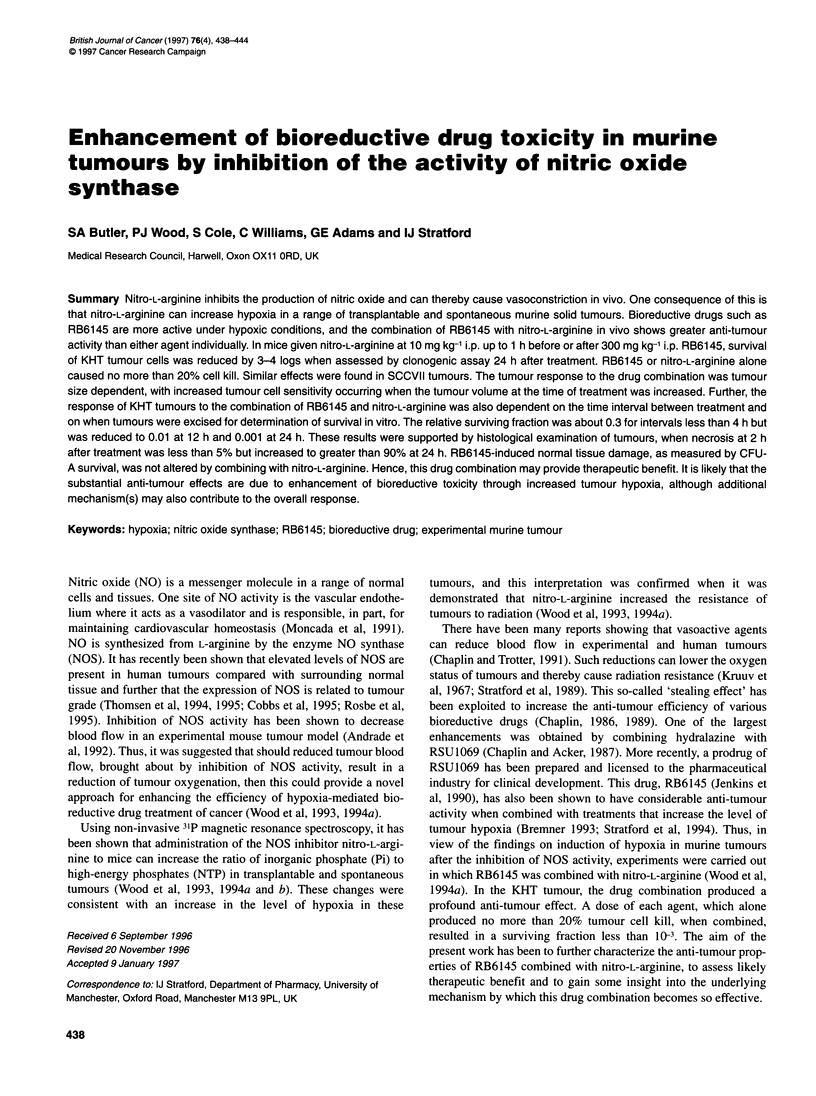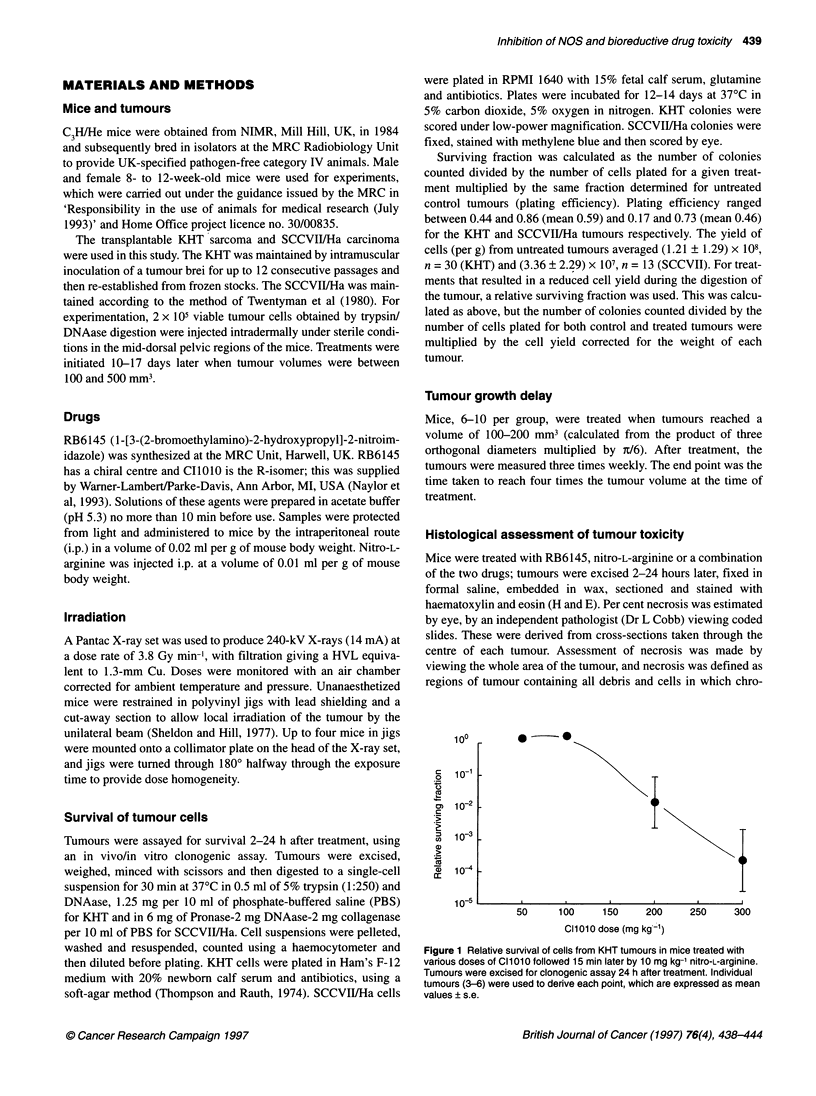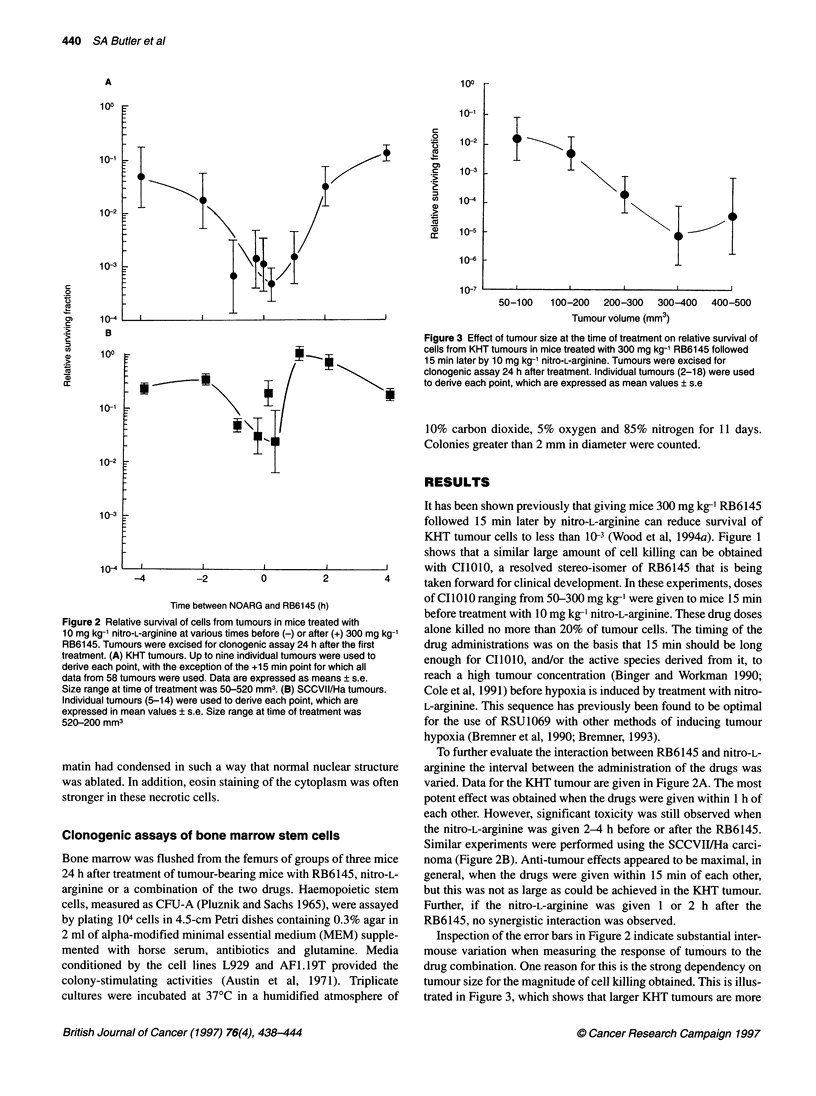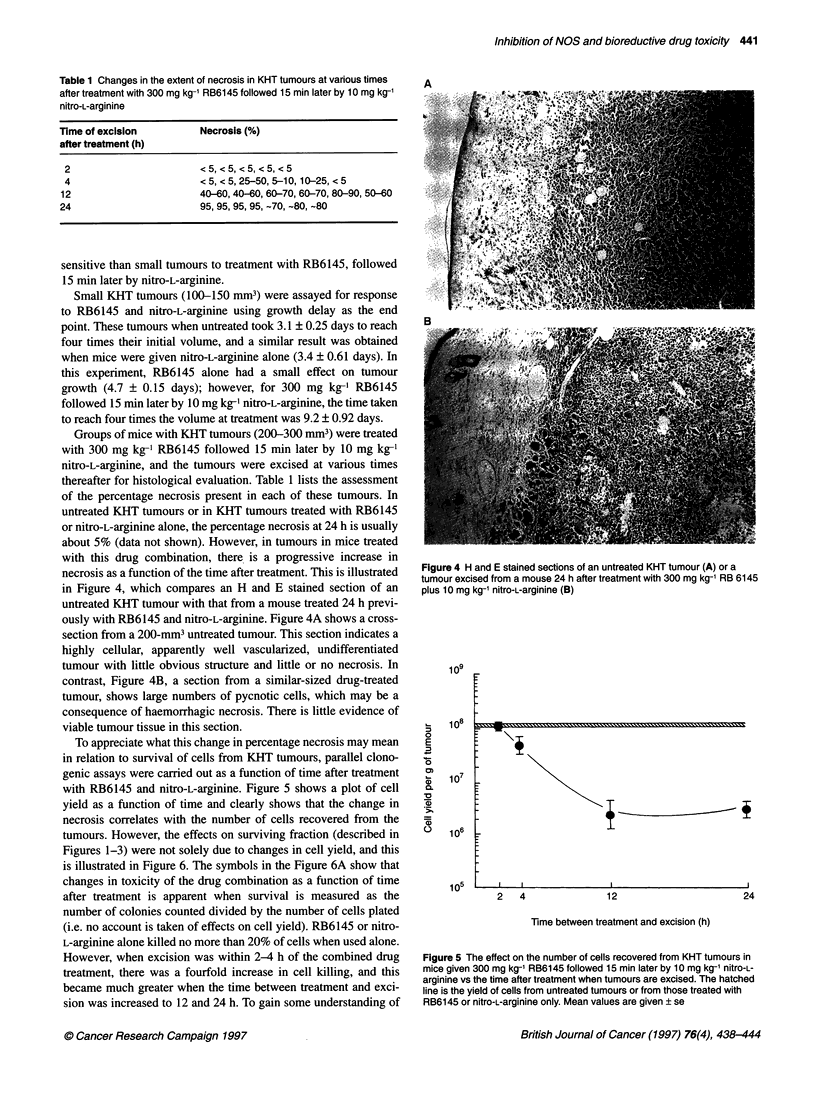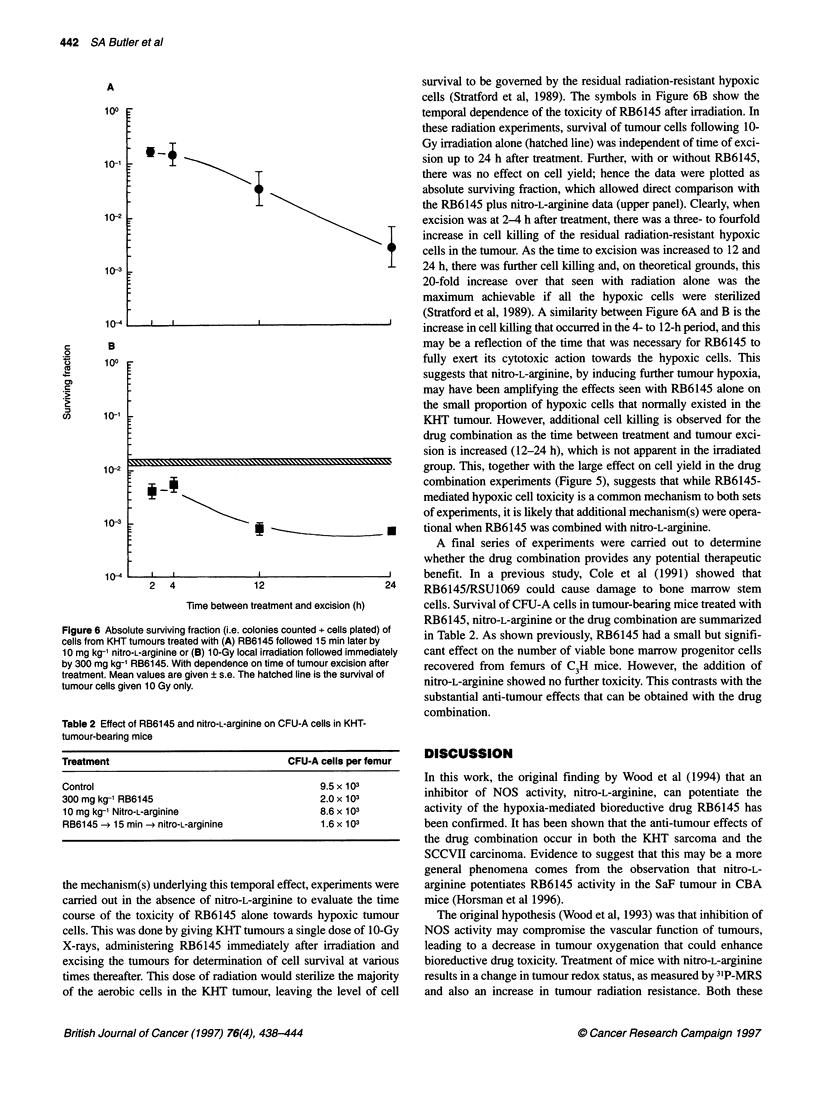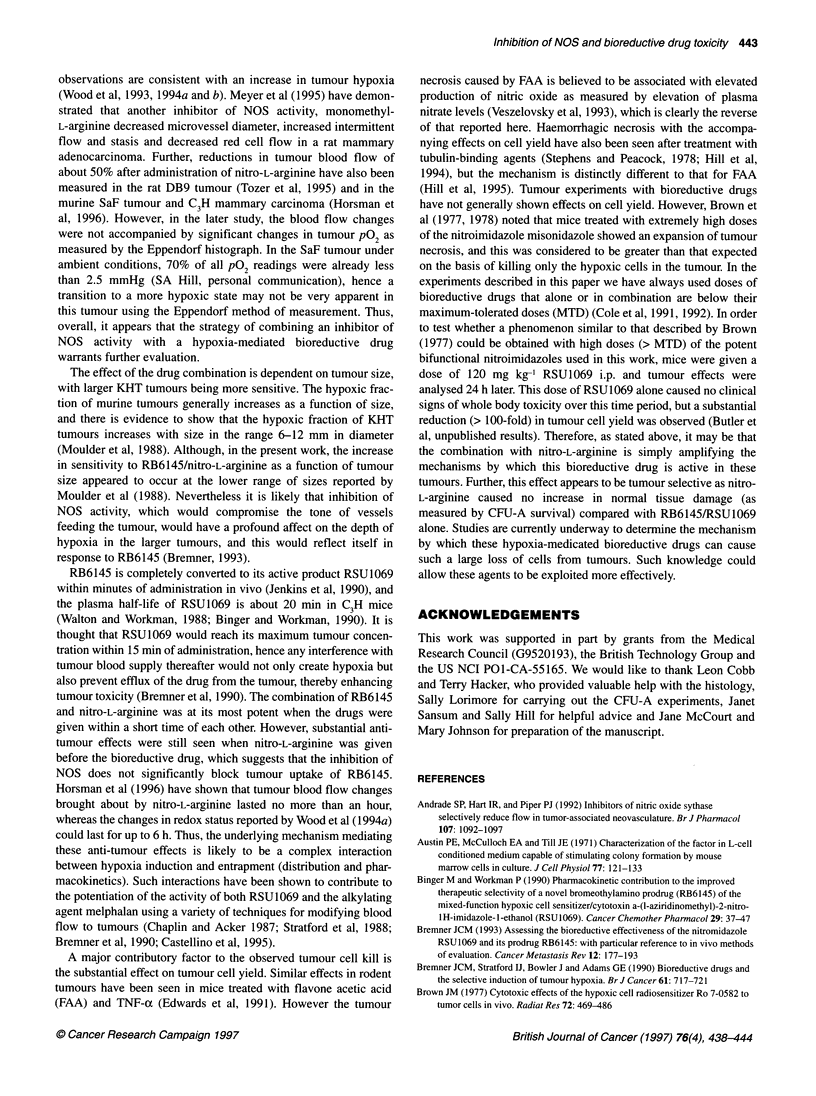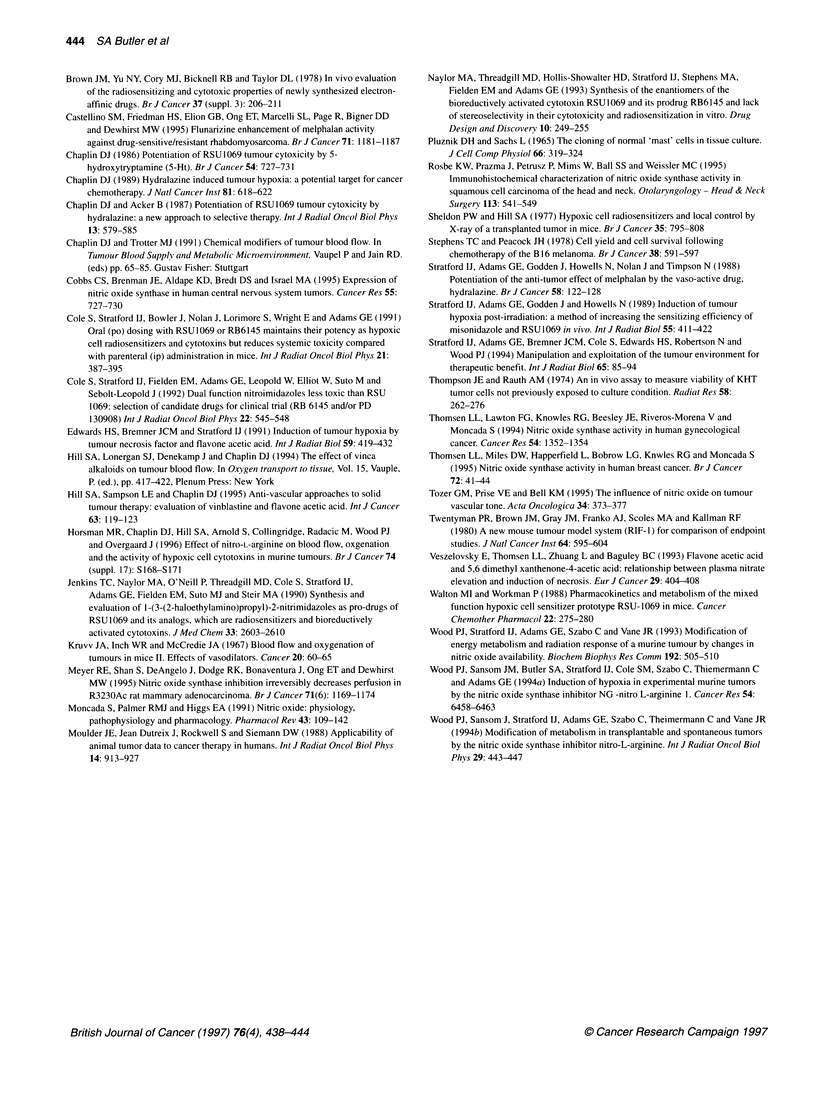# Enhancement of bioreductive drug toxicity in murine tumours by inhibition of the activity of nitric oxide synthase.

**DOI:** 10.1038/bjc.1997.407

**Published:** 1997

**Authors:** S. A. Butler, P. J. Wood, S. Cole, C. Williams, G. E. Adams, I. J. Stratford

**Affiliations:** Medical Research Council, Harwell, UK.

## Abstract

**Images:**


British Journal of Cancer (1997) 76(4), 438–444
© 1997 Cancer Research Campaign

### Enhancement of bioreductive drug toxicity in murine tumours by inhibition of the activity of nitric oxide synthase

**SA Butler, PJ Wood, S Cole, C Williams, GE Adams and IJ Stratford**

Medical Research Council, Harwell, Oxon OX11 0RD, UK

**Summary** Nitro-L-arginine inhibits the production of nitric oxide and can thereby cause vasoconstriction in vivo. One consequence of this is that nitro-L-arginine can increase hypoxia in a range of transplantable and spontaneous murine solid tumours. Bioreductive drugs such as RB6145 are more active under hypoxic conditions, and the combination of RB6145 with nitro-L-arginine in vivo shows greater anti-tumour activity than either agent individually. In mice given nitro-L-arginine at 10 mg $kg^{-1}$ i.p. up to 1 h before or after 300 mg $kg^{-1}$ i.p. RB6145, survival of KHT tumour cells was reduced by 3–4 logs when assessed by clonogenic assay 24 h after treatment. RB6145 or nitro-L-arginine alone caused no more than 20% cell kill. Similar effects were found in SCCVII tumours. The tumour response to the drug combination was tumour size dependent, with increased tumour cell sensitivity occurring when the tumour volume at the time of treatment was increased. Further, the response of KHT tumours to the combination of RB6145 and nitro-L-arginine was also dependent on the time interval between treatment and on when tumours were excised for determination of survival in vitro. The relative surviving fraction was about 0.3 for intervals less than 4 h but was reduced to 0.01 at 12 h and 0.001 at 24 h. These results were supported by histological examination of tumours, when necrosis at 2 h after treatment was less than 5% but increased to greater than 90% at 24 h. RB6145-induced normal tissue damage, as measured by CFU-A survival, was not altered by combining with nitro-L-arginine. Hence, this drug combination may provide therapeutic benefit. It is likely that the substantial anti-tumour effects are due to enhancement of bioreductive toxicity through increased tumour hypoxia, although additional mechanism(s) may also contribute to the overall response.

**Keywords:** hypoxia; nitric oxide synthase; RB6145; bioreductive drug; experimental murine tumour

Nitric oxide (NO) is a messenger molecule in a range of normal cells and tissues. One site of NO activity is the vascular endothelium where it acts as a vasodilator and is responsible, in part, for maintaining cardiovascular homeostasis (Moncada et al, 1991). NO is synthesized from L-arginine by the enzyme NO synthase (NOS). It has recently been shown that elevated levels of NOS are present in human tumours compared with surrounding normal tissue and further that the expression of NOS is related to tumour grade (Thomsen et al, 1994, 1995; Cobbs et al, 1995; Rosbe et al, 1995). Inhibition of NOS activity has been shown to decrease blood flow in an experimental mouse tumour model (Andrade et al, 1992). Thus, it was suggested that should reduced tumour blood flow, brought about by inhibition of NOS activity, result in a reduction of tumour oxygenation, then this could provide a novel approach for enhancing the efficiency of hypoxia-mediated bioreductive drug treatment of cancer (Wood et al, 1993, 1994*a*).

Using non-invasive $^{31}P$ magnetic resonance spectroscopy, it has been shown that administration of the NOS inhibitor nitro-L-arginine to mice can increase the ratio of inorganic phosphate (Pi) to high-energy phosphates (NTP) in transplantable and spontaneous tumours (Wood et al, 1993, 1994*a* and *b*). These changes were consistent with an increase in the level of hypoxia in these tumours, and this interpretation was confirmed when it was demonstrated that nitro-L-arginine increased the resistance of tumours to radiation (Wood et al, 1993, 1994*a*).

There have been many reports showing that vasoactive agents can reduce blood flow in experimental and human tumours (Chaplin and Trotter, 1991). Such reductions can lower the oxygen status of tumours and thereby cause radiation resistance (Kruuv et al, 1967; Stratford et al, 1989). This so-called 'stealing effect' has been exploited to increase the anti-tumour efficiency of various bioreductive drugs (Chaplin, 1986, 1989). One of the largest enhancements was obtained by combining hydralazine with RSU1069 (Chaplin and Acker, 1987). More recently, a prodrug of RSU1069 has been prepared and licensed to the pharmaceutical industry for clinical development. This drug, RB6145 (Jenkins et al, 1990), has also been shown to have considerable anti-tumour activity when combined with treatments that increase the level of tumour hypoxia (Bremner 1993; Stratford et al, 1994). Thus, in view of the findings on induction of hypoxia in murine tumours after the inhibition of NOS activity, experiments were carried out in which RB6145 was combined with nitro-L-arginine (Wood et al, 1994*a*). In the KHT tumour, the drug combination produced a profound anti-tumour effect. A dose of each agent, which alone produced no more than 20% tumour cell kill, when combined, resulted in a surviving fraction less than $10^{-3}$. The aim of the present work has been to further characterize the anti-tumour properties of RB6145 combined with nitro-L-arginine, to assess likely therapeutic benefit and to gain some insight into the underlying mechanism by which this drug combination becomes so effective.

*Received 6 September 1996*
*Revised 20 November 1996*
*Accepted 9 January 1997*

*Correspondence to:* IJ Stratford, Department of Pharmacy, University of Manchester, Oxford Road, Manchester M13 9PL, UK

### MATERIALS AND METHODS

### Mice and tumours

$C_3H$/He mice were obtained from NIMR, Mill Hill, UK, in 1984 and subsequently bred in isolators at the MRC Radiobiology Unit to provide UK-specified pathogen-free category IV animals. Male and female 8- to 12-week-old mice were used for experiments, which were carried out under the guidance issued by the MRC in 'Responsibility in the use of animals for medical research (July 1993)' and Home Office project licence no. 30/00835.

The transplantable KHT sarcoma and SCCVII/Ha carcinoma were used in this study. The KHT was maintained by intramuscular inoculation of a tumour brei for up to 12 consecutive passages and then re-established from frozen stocks. The SCCVII/Ha was maintained according to the method of Twentyman et al (1980). For experimentation, $2 \times 10^5$ viable tumour cells obtained by trypsin/DNAase digestion were injected intradermally under sterile conditions in the mid-dorsal pelvic regions of the mice. Treatments were initiated 10–17 days later when tumour volumes were between 100 and 500 $mm^3$.

### Drugs

RB6145 (1-[3-(2-bromoethylamino)-2-hydroxypropyl]-2-nitroimidazole) was synthesized at the MRC Unit, Harwell, UK. RB6145 has a chiral centre and CI1010 is the R-isomer; this was supplied by Warner-Lambert/Parke-Davis, Ann Arbor, MI, USA (Naylor et al, 1993). Solutions of these agents were prepared in acetate buffer (pH 5.3) no more than 10 min before use. Samples were protected from light and administered to mice by the intraperitoneal route (i.p.) in a volume of 0.02 ml per g of mouse body weight. Nitro-L-arginine was injected i.p. at a volume of 0.01 ml per g of mouse body weight.

### Irradiation

A Pantac X-ray set was used to produce 240-kV X-rays (14 mA) at a dose rate of 3.8 Gy $min^{-1}$, with filtration giving a HVL equivalent to 1.3-mm Cu. Doses were monitored with an air chamber corrected for ambient temperature and pressure. Unanaesthetized mice were restrained in polyvinyl jigs with lead shielding and a cut-away section to allow local irradiation of the tumour by the unilateral beam (Sheldon and Hill, 1977). Up to four mice in jigs were mounted onto a collimator plate on the head of the X-ray set, and jigs were turned through 180° halfway through the exposure time to provide dose homogeneity.

### Survival of tumour cells

Tumours were assayed for survival 2–24 h after treatment, using an in vivo/in vitro clonogenic assay. Tumours were excised, weighed, minced with scissors and then digested to a single-cell suspension for 30 min at 37°C in 0.5 ml of 5% trypsin (1:250) and DNAase, 1.25 mg per 10 ml of phosphate-buffered saline (PBS) for KHT and in 6 mg of Pronase-2 mg DNAase-2 mg collagenase per 10 ml of PBS for SCCVII/Ha. Cell suspensions were pelleted, washed and resuspended, counted using a haemocytometer and then diluted before plating. KHT cells were plated in Ham's F-12 medium with 20% newborn calf serum and antibiotics, using a soft-agar method (Thompson and Rauth, 1974). SCCVII/Ha cells were plated in RPMI 1640 with 15% fetal calf serum, glutamine and antibiotics. Plates were incubated for 12–14 days at 37°C in 5% carbon dioxide, 5% oxygen in nitrogen. KHT colonies were scored under low-power magnification. SCCVII/Ha colonies were fixed, stained with methylene blue and then scored by eye.

Surviving fraction was calculated as the number of colonies counted divided by the number of cells plated for a given treatment multiplied by the same fraction determined for untreated control tumours (plating efficiency). Plating efficiency ranged between 0.44 and 0.86 (mean 0.59) and 0.17 and 0.73 (mean 0.46) for the KHT and SCCVII/Ha tumours respectively. The yield of cells (per g) from untreated tumours averaged $(1.21 \pm 1.29) \times 10^8$, $n = 30$ (KHT) and $(3.36 \pm 2.29) \times 10^7$, $n = 13$ (SCCVII). For treatments that resulted in a reduced cell yield during the digestion of the tumour, a relative surviving fraction was used. This was calculated as above, but the number of colonies counted divided by the number of cells plated for both control and treated tumours were multiplied by the cell yield corrected for the weight of each tumour.

### Tumour growth delay

Mice, 6–10 per group, were treated when tumours reached a volume of 100–200 $mm^3$ (calculated from the product of three orthogonal diameters multiplied by $\pi/6$). After treatment, the tumours were measured three times weekly. The end point was the time taken to reach four times the tumour volume at the time of treatment.

### Histological assessment of tumour toxicity

Mice were treated with RB6145, nitro-L-arginine or a combination of the two drugs; tumours were excised 2–24 hours later, fixed in formal saline, embedded in wax, sectioned and stained with haematoxylin and eosin (H and E). Per cent necrosis was estimated by eye, by an independent pathologist (Dr L Cobb) viewing coded slides. These were derived from cross-sections taken through the centre of each tumour. Assessment of necrosis was made by viewing the whole area of the tumour, and necrosis was defined as regions of tumour containing all debris and cells in which chro-

**Figure 1** Relative survival of cells from KHT tumours in mice treated with various doses of CI1010 followed 15 min later by 10 mg $kg^{-1}$ nitro-L-arginine. Tumours were excised for clonogenic assay 24 h after treatment. Individual tumours (3–6) were used to derive each point, which are expressed as mean values ± s.e.

© Cancer Research Campaign 1997

**Figure 2** Relative survival of cells from tumours in mice treated with 10 mg kg$^{-1}$ nitro-L-arginine at various times before (–) or after (+) 300 mg kg$^{-1}$ RB6145. Tumours were excised for clonogenic assay 24 h after the first treatment. (**A**) KHT tumours. Up to nine individual tumours were used to derive each point, with the exception of the +15 min point for which all data from 58 tumours were used. Data are expressed as means ± s.e. Size range at time of treatment was 50–520 mm$^3$. (**B**) SCCVII/Ha tumours. Individual tumours (5–14) were used to derive each point, which are expressed in mean values ± s.e. Size range at time of treatment was 520–200 mm$^3$

**Figure 3** Effect of tumour size at the time of treatment on relative survival of cells from KHT tumours in mice treated with 300 mg kg$^{-1}$ RB6145 followed 15 min later by 10 mg kg$^{-1}$ nitro-L-arginine. Tumours were excised for clonogenic assay 24 h after treatment. Individual tumours (2–18) were used to derive each point, which are expressed as mean values ± s.e matin had condensed in such a way that normal nuclear structure was ablated. In addition, eosin staining of the cytoplasm was often stronger in these necrotic cells.

### Clonogenic assays of bone marrow stem cells

Bone marrow was flushed from the femurs of groups of three mice 24 h after treatment of tumour-bearing mice with RB6145, nitro-L-arginine or a combination of the two drugs. Haemopoietic stem cells, measured as CFU-A (Pluznik and Sachs 1965), were assayed by plating $10^4$ cells in 4.5-cm Petri dishes containing 0.3% agar in 2 ml of alpha-modified minimal essential medium (MEM) supplemented with horse serum, antibiotics and glutamine. Media conditioned by the cell lines L929 and AF1.19T provided the colony-stimulating activities (Austin et al, 1971). Triplicate cultures were incubated at 37°C in a humidified atmosphere of 10% carbon dioxide, 5% oxygen and 85% nitrogen for 11 days. Colonies greater than 2 mm in diameter were counted.

### RESULTS

It has been shown previously that giving mice 300 mg kg$^{-1}$ RB6145 followed 15 min later by nitro-L-arginine can reduce survival of KHT tumour cells to less than $10^{-3}$ (Wood et al, 1994*a*). Figure 1 shows that a similar large amount of cell killing can be obtained with CI1010, a resolved stereo-isomer of RB6145 that is being taken forward for clinical development. In these experiments, doses of CI1010 ranging from 50–300 mg kg$^{-1}$ were given to mice 15 min before treatment with 10 mg kg$^{-1}$ nitro-L-arginine. These drug doses alone killed no more than 20% of tumour cells. The timing of the drug administrations was on the basis that 15 min should be long enough for CI1010, and/or the active species derived from it, to reach a high tumour concentration (Binger and Workman 1990; Cole et al, 1991) before hypoxia is induced by treatment with nitro-L-arginine. This sequence has previously been found to be optimal for the use of RSU1069 with other methods of inducing tumour hypoxia (Bremner et al, 1990; Bremner, 1993).

To further evaluate the interaction between RB6145 and nitro-L-arginine the interval between the administration of the drugs was varied. Data for the KHT tumour are given in Figure 2A. The most potent effect was obtained when the drugs were given within 1 h of each other. However, significant toxicity was still observed when the nitro-L-arginine was given 2–4 h before or after the RB6145. Similar experiments were performed using the SCCVII/Ha carcinoma (Figure 2B). Anti-tumour effects appeared to be maximal, in general, when the drugs were given within 15 min of each other, but this was not as large as could be achieved in the KHT tumour. Further, if the nitro-L-arginine was given 1 or 2 h after the RB6145, no synergistic interaction was observed.

Inspection of the error bars in Figure 2 indicate substantial inter-mouse variation when measuring the response of tumours to the drug combination. One reason for this is the strong dependency on tumour size for the magnitude of cell killing obtained. This is illustrated in Figure 3, which shows that larger KHT tumours are more

© Cancer Research Campaign 1997

**Table 1** Changes in the extent of necrosis in KHT tumours at various times after treatment with 300 mg $kg^{-1}$ RB6145 followed 15 min later by 10 mg $kg^{-1}$ nitro-L-arginine

| Time of excision after treatment (h) | Necrosis (%) |
|---|---|
| 2 | < 5, < 5, < 5, < 5, < 5 |
| 4 | < 5, < 5, 25–50, 5–10, 10–25, < 5 |
| 12 | 40–60, 40–60, 60–70, 60–70, 80–90, 50–60 |
| 24 | 95, 95, 95, 95, ~70, ~80, ~80 |

sensitive than small tumours to treatment with RB6145, followed 15 min later by nitro-L-arginine.

Small KHT tumours (100–150 $mm^3$) were assayed for response to RB6145 and nitro-L-arginine using growth delay as the end point. These tumours when untreated took 3.1 ± 0.25 days to reach four times their initial volume, and a similar result was obtained when mice were given nitro-L-arginine alone (3.4 ± 0.61 days). In this experiment, RB6145 alone had a small effect on tumour growth (4.7 ± 0.15 days); however, for 300 mg $kg^{-1}$ RB6145 followed 15 min later by 10 mg $kg^{-1}$ nitro-L-arginine, the time taken to reach four times the volume at treatment was 9.2 ± 0.92 days.

Groups of mice with KHT tumours (200–300 $mm^3$) were treated with 300 mg $kg^{-1}$ RB6145 followed 15 min later by 10 mg $kg^{-1}$ nitro-L-arginine, and the tumours were excised at various times thereafter for histological evaluation. Table 1 lists the assessment of the percentage necrosis present in each of these tumours. In untreated KHT tumours or in KHT tumours treated with RB6145 or nitro-L-arginine alone, the percentage necrosis at 24 h is usually about 5% (data not shown). However, in tumours in mice treated with this drug combination, there is a progressive increase in necrosis as a function of the time after treatment. This is illustrated in Figure 4, which compares an H and E stained section of an untreated KHT tumour with that from a mouse treated 24 h previously with RB6145 and nitro-L-arginine. Figure 4A shows a cross-section from a 200-$mm^3$ untreated tumour. This section indicates a highly cellular, apparently well vascularized, undifferentiated tumour with little obvious structure and little or no necrosis. In contrast, Figure 4B, a section from a similar-sized drug-treated tumour, shows large numbers of pycnotic cells, which may be a consequence of haemorrhagic necrosis. There is little evidence of viable tumour tissue in this section.

To appreciate what this change in percentage necrosis may mean in relation to survival of cells from KHT tumours, parallel clonogenic assays were carried out as a function of time after treatment with RB6145 and nitro-L-arginine. Figure 5 shows a plot of cell yield as a function of time and clearly shows that the change in necrosis correlates with the number of cells recovered from the tumours. However, the effects on surviving fraction (described in Figures 1–3) were not solely due to changes in cell yield, and this is illustrated in Figure 6. The symbols in the Figure 6A show that changes in toxicity of the drug combination as a function of time after treatment is apparent when survival is measured as the number of colonies counted divided by the number of cells plated (i.e. no account is taken of effects on cell yield). RB6145 or nitro-L-arginine alone killed no more than 20% of cells when used alone. However, when excision was within 2–4 h of the combined drug treatment, there was a fourfold increase in cell killing, and this became much greater when the time between treatment and excision was increased to 12 and 24 h. To gain some understanding of

**Figure 4** H and E stained sections of an untreated KHT tumour (**A**) or a tumour excised from a mouse 24 h after treatment with 300 mg $kg^{-1}$ RB 6145 plus 10 mg $kg^{-1}$ nitro-L-arginine (**B**)

**Figure 5** The effect on the number of cells recovered from KHT tumours in mice given 300 mg $kg^{-1}$ RB6145 followed 15 min later by 10 mg $kg^{-1}$ nitro-L-arginine vs the time after treatment when tumours are excised. The hatched line is the yield of cells from untreated tumours or from those treated with RB6145 or nitro-L-arginine only. Mean values are given ± se

© Cancer Research Campaign 1997

**Figure 6** Absolute surviving fraction (i.e. colonies counted ÷ cells plated) of cells from KHT tumours treated with (**A**) RB6145 followed 15 min later by 10 mg $kg^{-1}$ nitro-L-arginine or (**B**) 10-Gy local irradiation followed immediately by 300 mg $kg^{-1}$ RB6145. With dependence on time of tumour excision after treatment. Mean values are given ± s.e. The hatched line is the survival of tumour cells given 10 Gy only.

**Table 2** Effect of RB6145 and nitro-L-arginine on CFU-A cells in KHT-tumour-bearing mice

| Treatment | CFU-A cells per femur |
|---|---|
| Control | $9.5 \times 10^3$ |
| 300 mg $kg^{-1}$ RB6145 | $2.0 \times 10^3$ |
| 10 mg $kg^{-1}$ Nitro-L-arginine | $8.6 \times 10^3$ |
| RB6145 → 15 min → nitro-L-arginine | $1.6 \times 10^3$ |

the mechanism(s) underlying this temporal effect, experiments were carried out in the absence of nitro-L-arginine to evaluate the time course of the toxicity of RB6145 alone towards hypoxic tumour cells. This was done by giving KHT tumours a single dose of 10-Gy X-rays, administering RB6145 immediately after irradiation and excising the tumours for determination of cell survival at various times thereafter. This dose of radiation would sterilize the majority of the aerobic cells in the KHT tumour, leaving the level of cell survival to be governed by the residual radiation-resistant hypoxic cells (Stratford et al, 1989). The symbols in Figure 6B show the temporal dependence of the toxicity of RB6145 after irradiation. In these radiation experiments, survival of tumour cells following 10-Gy irradiation alone (hatched line) was independent of time of excision up to 24 h after treatment. Further, with or without RB6145, there was no effect on cell yield; hence the data were plotted as absolute surviving fraction, which allowed direct comparison with the RB6145 plus nitro-L-arginine data (upper panel). Clearly, when excision was at 2–4 h after treatment, there was a three- to fourfold increase in cell killing of the residual radiation-resistant hypoxic cells in the tumour. As the time to excision was increased to 12 and 24 h, there was further cell killing and, on theoretical grounds, this 20-fold increase over that seen with radiation alone was the maximum achievable if all the hypoxic cells were sterilized (Stratford et al, 1989). A similarity between Figure 6A and B is the increase in cell killing that occurred in the 4- to 12-h period, and this may be a reflection of the time that was necessary for RB6145 to fully exert its cytotoxic action towards the hypoxic cells. This suggests that nitro-L-arginine, by inducing further tumour hypoxia, may have been amplifying the effects seen with RB6145 alone on the small proportion of hypoxic cells that normally existed in the KHT tumour. However, additional cell killing is observed for the drug combination as the time between treatment and tumour excision is increased (12–24 h), which is not apparent in the irradiated group. This, together with the large effect on cell yield in the drug combination experiments (Figure 5), suggests that while RB6145-mediated hypoxic cell toxicity is a common mechanism to both sets of experiments, it is likely that additional mechanism(s) were operational when RB6145 was combined with nitro-L-arginine.

A final series of experiments were carried out to determine whether the drug combination provides any potential therapeutic benefit. In a previous study, Cole et al (1991) showed that RB6145/RSU1069 could cause damage to bone marrow stem cells. Survival of CFU-A cells in tumour-bearing mice treated with RB6145, nitro-L-arginine or the drug combination are summarized in Table 2. As shown previously, RB6145 had a small but significant effect on the number of viable bone marrow progenitor cells recovered from femurs of $C_3H$ mice. However, the addition of nitro-L-arginine showed no further toxicity. This contrasts with the substantial anti-tumour effects that can be obtained with the drug combination.

### DISCUSSION

In this work, the original finding by Wood et al (1994) that an inhibitor of NOS activity, nitro-L-arginine, can potentiate the activity of the hypoxia-mediated bioreductive drug RB6145 has been confirmed. It has been shown that the anti-tumour effects of the drug combination occur in both the KHT sarcoma and the SCCVII carcinoma. Evidence to suggest that this may be a more general phenomena comes from the observation that nitro-L-arginine potentiates RB6145 activity in the SaF tumour in CBA mice (Horsman et al 1996).

The original hypothesis (Wood et al, 1993) was that inhibition of NOS activity may compromise the vascular function of tumours, leading to a decrease in tumour oxygenation that could enhance bioreductive drug toxicity. Treatment of mice with nitro-L-arginine results in a change in tumour redox status, as measured by $^{31}$P-MRS and also an increase in tumour radiation resistance. Both these

© Cancer Research Campaign 1997

observations are consistent with an increase in tumour hypoxia (Wood et al, 1993, 1994*a* and *b*). Meyer et al (1995) have demonstrated that another inhibitor of NOS activity, monomethyl-L-arginine decreased microvessel diameter, increased intermittent flow and stasis and decreased red cell flow in a rat mammary adenocarcinoma. Further, reductions in tumour blood flow of about 50% after administration of nitro-L-arginine have also been measured in the rat DB9 tumour (Tozer et al, 1995) and in the murine SaF tumour and $C_3H$ mammary carcinoma (Horsman et al, 1996). However, in the later study, the blood flow changes were not accompanied by significant changes in tumour $pO_2$ as measured by the Eppendorf histograph. In the SaF tumour under ambient conditions, 70% of all $pO_2$ readings were already less than 2.5 mmHg (SA Hill, personal communication), hence a transition to a more hypoxic state may not be very apparent in this tumour using the Eppendorf method of measurement. Thus, overall, it appears that the strategy of combining an inhibitor of NOS activity with a hypoxia-mediated bioreductive drug warrants further evaluation.

The effect of the drug combination is dependent on tumour size, with larger KHT tumours being more sensitive. The hypoxic fraction of murine tumours generally increases as a function of size, and there is evidence to show that the hypoxic fraction of KHT tumours increases with size in the range 6–12 mm in diameter (Moulder et al, 1988). Although, in the present work, the increase in sensitivity to RB6145/nitro-L-arginine as a function of tumour size appeared to occur at the lower range of sizes reported by Moulder et al (1988). Nevertheless it is likely that inhibition of NOS activity, which would compromise the tone of vessels feeding the tumour, would have a profound affect on the depth of hypoxia in the larger tumours, and this would reflect itself in response to RB6145 (Bremner, 1993).

RB6145 is completely converted to its active product RSU1069 within minutes of administration in vivo (Jenkins et al, 1990), and the plasma half-life of RSU1069 is about 20 min in $C_3H$ mice (Walton and Workman, 1988; Binger and Workman, 1990). It is thought that RSU1069 would reach its maximum tumour concentration within 15 min of administration, hence any interference with tumour blood supply thereafter would not only create hypoxia but also prevent efflux of the drug from the tumour, thereby enhancing tumour toxicity (Bremner et al, 1990). The combination of RB6145 and nitro-L-arginine was at its most potent when the drugs were given within a short time of each other. However, substantial anti-tumour effects were still seen when nitro-L-arginine was given before the bioreductive drug, which suggests that the inhibition of NOS does not significantly block tumour uptake of RB6145. Horsman et al (1996) have shown that tumour blood flow changes brought about by nitro-L-arginine lasted no more than an hour, whereas the changes in redox status reported by Wood et al (1994*a*) could last for up to 6 h. Thus, the underlying mechanism mediating these anti-tumour effects is likely to be a complex interaction between hypoxia induction and entrapment (distribution and pharmacokinetics). Such interactions have been shown to contribute to the potentiation of the activity of both RSU1069 and the alkylating agent melphalan using a variety of techniques for modifying blood flow to tumours (Chaplin and Acker 1987; Stratford et al, 1988; Bremner et al, 1990; Castellino et al, 1995).

A major contributory factor to the observed tumour cell kill is the substantial effect on tumour cell yield. Similar effects in rodent tumours have been seen in mice treated with flavone acetic acid (FAA) and TNF-α (Edwards et al, 1991). However the tumour necrosis caused by FAA is believed to be associated with elevated production of nitric oxide as measured by elevation of plasma nitrate levels (Veszelovsky et al, 1993), which is clearly the reverse of that reported here. Haemorrhagic necrosis with the accompanying effects on cell yield have also been seen after treatment with tubulin-binding agents (Stephens and Peacock, 1978; Hill et al, 1994), but the mechanism is distinctly different to that for FAA (Hill et al, 1995). Tumour experiments with bioreductive drugs have not generally shown effects on cell yield. However, Brown et al (1977, 1978) noted that mice treated with extremely high doses of the nitroimidazole misonidazole showed an expansion of tumour necrosis, and this was considered to be greater than that expected on the basis of killing only the hypoxic cells in the tumour. In the experiments described in this paper we have always used doses of bioreductive drugs that alone or in combination are below their maximum-tolerated doses (MTD) (Cole et al, 1991, 1992). In order to test whether a phenomenon similar to that described by Brown (1977) could be obtained with high doses (> MTD) of the potent bifunctional nitroimidazoles used in this work, mice were given a dose of 120 mg $kg^{-1}$ RSU1069 i.p. and tumour effects were analysed 24 h later. This dose of RSU1069 alone caused no clinical signs of whole body toxicity over this time period, but a substantial reduction (> 100-fold) in tumour cell yield was observed (Butler et al, unpublished results). Therefore, as stated above, it may be that the combination with nitro-L-arginine is simply amplifying the mechanisms by which this bioreductive drug is active in these tumours. Further, this effect appears to be tumour selective as nitro-L-arginine caused no increase in normal tissue damage (as measured by CFU-A survival) compared with RB6145/RSU1069 alone. Studies are currently underway to determine the mechanism by which these hypoxia-medicated bioreductive drugs can cause such a large loss of cells from tumours. Such knowledge could allow these agents to be exploited more effectively.

### ACKNOWLEDGEMENTS

This work was supported in part by grants from the Medical Research Council (G9520193), the British Technology Group and the US NCI PO1-CA-55165. We would like to thank Leon Cobb and Terry Hacker, who provided valuable help with the histology, Sally Lorimore for carrying out the CFU-A experiments, Janet Sansum and Sally Hill for helpful advice and Jane McCourt and Mary Johnson for preparation of the manuscript.

### REFERENCES

Andrade SP, Hart IR, and Piper PJ (1992) Inhibitors of nitric oxide sythase selectively reduce flow in tumor-associated neovasculature. *Br J Pharmacol* **107**: 1092–1097

Austin PE, McCulloch EA and Till JE (1971) Characterization of the factor in L-cell conditioned medium capable of stimulating colony formation by mouse marrow cells in culture. *J Cell Physiol* **77**: 121–133

Binger M and Workman P (1990) Pharmacokinetic contribution to the improved therapeutic selectivity of a novel bromeothylamino prodrug (RB6145) of the mixed-function hypoxic cell sensitizer/cytotoxin a-(l-aziridinomethyl)-2-nitro-1H-imidazole-1-ethanol (RSU1069). *Cancer Chemother Pharmacol* **29**: 37–47

Bremner JCM (1993) Assessing the bioreductive effectiveness of the nitromidazole RSU1069 and its prodrug RB6145: with particular reference to in vivo methods of evaluation. *Cancer Metastasis Rev* **12**: 177–193

Bremner JCM, Stratford IJ, Bowler J and Adams GE (1990) Bioreductive drugs and the selective induction of tumour hypoxia. *Br J Cancer* **61**: 717–721

Brown JM (1977) Cytotoxic effects of the hypoxic cell radiosensitizer Ro 7-0582 to tumor cells in vivo. *Radiat Res* **72**: 469–486

© Cancer Research Campaign 1997

Brown JM, Yu NY, Cory MJ, Bicknell RB and Taylor DL (1978) In vivo evaluation of the radiosensitizing and cytotoxic properties of newly synthesized electron-affinic drugs. *Br J Cancer* **37** (suppl. 3): 206–211

Castellino SM, Friedman HS, Elion GB, Ong ET, Marcelli SL, Page R, Bigner DD and Dewhirst MW (1995) Flunarizine enhancement of melphalan activity against drug-sensitive/resistant rhabdomyosarcoma. *Br J Cancer* **71**: 1181–1187

Chaplin DJ (1986) Potentiation of RSU1069 tumour cytoxicity by 5-hydroxytryptamine (5-Ht). *Br J Cancer* **54**: 727–731

Chaplin DJ (1989) Hydralazine induced tumour hypoxia: a potential target for cancer chemotherapy. *J Natl Cancer Inst* **81**: 618–622

Chaplin DJ and Acker B (1987) Potentiation of RSU1069 tumour cytoxicity by hydralazine: a new approach to selective therapy. *Int J Radial Oncol Biol Phys* **13**: 579–585

Chaplin DJ and Trotter MJ (1991) Chemical modifiers of tumour blood flow. In *Tumour Blood Supply and Metabolic Microenvironment,* Vaupel P and Jain RD. (eds) pp. 65–85. Gustav Fisher: Stuttgart

Cobbs CS, Brenman JE, Aldape KD, Bredt DS and Israel MA (1995) Expression of nitric oxide synthase in human central nervous system tumors. *Cancer Res* **55**: 727–730

Cole S, Stratford IJ, Bowler J, Nolan J, Lorimore S, Wright E and Adams GE (1991) Oral (po) dosing with RSU1069 or RB6145 maintains their potency as hypoxic cell radiosensitizers and cytotoxins but reduces systemic toxicity compared with parenteral (ip) administration in mice. *Int J Radiat Oncol Biol Phys* **21**: 387–395

Cole S, Stratford IJ, Fielden EM, Adams GE, Leopold W, Elliot W, Suto M and Sebolt-Leopold J (1992) Dual function nitroimidazoles less toxic than RSU 1069: selection of candidate drugs for clinical trial (RB 6145 and/or PD 130908) *Int J Radiat Oncol Biol Phys* **22**: 545–548

Edwards HS, Bremner JCM and Stratford IJ (1991) Induction of tumour hypoxia by tumour necrosis factor and flavone acetic acid. *Int J Radiat Biol* **59**: 419–432

Hill SA, Lonergan SJ, Denekamp J and Chaplin DJ (1994) The effect of vinca alkaloids on tumour blood flow. In *Oxygen transport to tissue,* Vol. 15, Vauple, P. (ed.), pp. 417–422, Plenum Press: New York

Hill SA, Sampson LE and Chaplin DJ (1995) Anti-vascular approaches to solid tumour therapy: evaluation of vinblastine and flavone acetic acid. *Int J Cancer* **63**: 119–123

Horsman MR, Chaplin DJ, Hill SA, Arnold S, Collingridge, Radacic M, Wood PJ and Overgaard J (1996) Effect of nitro-L-arginine on blood flow, oxgenation and the activity of hypoxic cell cytotoxins in murine tumours. *Br J Cancer* **74** (suppl. 17): S168–S171

Jenkins TC, Naylor MA, O'Neill P, Threadgill MD, Cole S, Stratford IJ, Adams GE, Fielden EM, Suto MJ and Steir MA (1990) Synthesis and evaluation of 1-(3-(2-haloethylamino)propyl)-2-nitrimidazoles as pro-drugs of RSU1069 and its analogs, which are radiosensitizers and bioreductively activated cytotoxins. *J Med Chem* **33**: 2603–2610

Kruvv JA, Inch WR and McCredie JA (1967) Blood flow and oxygenation of tumours in mice II. Effects of vasodilators. *Cancer* **20**: 60–65

Meyer RE, Shan S, DeAngelo J, Dodge RK, Bonaventura J, Ong ET and Dewhirst MW (1995) Nitric oxide synthase inhibition irreversibly decreases perfusion in R3230Ac rat mammary adenocarcinoma. *Br J Cancer* **71**(6): 1169–1174

Moncada S, Palmer RMJ and Higgs EA (1991) Nitric oxide: physiology, pathophysiology and pharmacology. *Pharmacol Rev* **43**: 109–142

Moulder JE, Jean Dutreix J, Rockwell S and Siemann DW (1988) Applicability of animal tumor data to cancer therapy in humans. *Int J Radiat Oncol Biol Phys* **14**: 913–927

Naylor MA, Threadgill MD, Hollis-Showalter HD, Stratford IJ, Stephens MA, Fielden EM and Adams GE (1993) Synthesis of the enantiomers of the bioreductively activated cytotoxin RSU1069 and its prodrug RB6145 and lack of stereoselectivity in their cytotoxicity and radiosensitization in vitro. *Drug Design and Discovery* **10**: 249–255

Pluznik DH and Sachs L (1965) The cloning of normal 'mast' cells in tissue culture. *J Cell Comp Physiol* **66**: 319–324

Rosbe KW, Prazma J, Petrusz P, Mims W, Ball SS and Weissler MC (1995) Immunohistochemical characterization of nitric oxide synthase activity in squamous cell carcinoma of the head and neck. *Otolaryngology – Head & Neck Surgery* **113**: 541–549

Sheldon PW and Hill SA (1977) Hypoxic cell radiosensitizers and local control by X-ray of a transplanted tumor in mice. *Br J Cancer* **35**: 795–808

Stephens TC and Peacock JH (1978) Cell yield and cell survival following chemotherapy of the B16 melanoma. *Br J Cancer* **38**: 591–597

Stratford IJ, Adams GE, Godden J, Howells N, Nolan J and Timpson N (1988) Potentiation of the anti-tumor effect of melphalan by the vaso-active drug, hydralazine. *Br J Cancer* **58**: 122–128

Stratford IJ, Adams GE, Godden J and Howells N (1989) Induction of tumour hypoxia post-irradiation: a method of increasing the sensitizing efficiency of misonidazole and RSU1069 *in vivo. Int J Radiat Biol* **55**: 411–422

Stratford IJ, Adams GE, Bremner JCM, Cole S, Edwards HS, Robertson N and Wood PJ (1994) Manipulation and exploitation of the tumour environment for therapeutic benefit. *Int J Radiat Biol* **65**: 85–94

Thompson JE and Rauth AM (1974) An in vivo assay to measure viability of KHT tumor cells not previously exposed to culture condition. *Radiat Res* **58**: 262–276

Thomsen LL, Lawton FG, Knowles RG, Beesley JE, Riveros-Morena V and Moncada S (1994) Nitric oxide synthase activity in human gynecological cancer. *Cancer Res* **54**: 1352–1354

Thomsen LL, Miles DW, Happerfield L, Bobrow LG, Knwles RG and Moncada S (1995) Nitric oxide synthase activity in human breast cancer. *Br J Cancer* **72**: 41–44

Tozer GM, Prise VE and Bell KM (1995) The influence of nitric oxide on tumour vascular tone. *Acta Oncologica* **34**: 373–377

Twentyman PR, Brown JM, Gray JM, Franko AJ, Scoles MA and Kallman RF (1980) A new mouse tumour model system (RIF-1) for comparison of endpoint studies. *J Natl Cancer Inst* **64**: 595–604

Veszelovsky E, Thomsen LL, Zhuang L and Baguley BC (1993) Flavone acetic acid and 5,6 dimethyl xanthenone-4-acetic acid: relationship between plasma nitrate elevation and induction of necrosis. *Eur J Cancer* **29**: 404–408

Walton MI and Workman P (1988) Pharmacokinetics and metabolism of the mixed function hypoxic cell sensitizer prototype RSU-1069 in mice. *Cancer Chemother Pharmacol* **22**: 275–280

Wood PJ, Stratford IJ, Adams GE, Szabo C and Vane JR (1993) Modification of energy metabolism and radiation response of a murine tumour by changes in nitric oxide availability. *Biochem Biophys Res Comm* **192**: 505–510

Wood PJ, Sansom JM, Butler SA, Stratford IJ, Cole SM, Szabo C, Thiemermann C and Adams GE (1994*a*) Induction of hypoxia in experimental murine tumors by the nitric oxide synthase inhibitor NG -nitro L-arginine 1. *Cancer Res* **54**: 6458–6463

Wood PJ, Sansom J, Stratford IJ, Adams GE, Szabo C, Theimermann C and Vane JR (1994*b*) Modification of metabolism in transplantable and spontaneous tumors by the nitric oxide synthase inhibitor nitro-L-arginine. *Int J Radiat Oncol Biol Phys* **29**: 443–447

 © Cancer Research Campaign 1997